# CBCT-Based Design of Patient-Specific 3D Bone Grafts for Periodontal Regeneration

**DOI:** 10.3390/jcm12155023

**Published:** 2023-07-30

**Authors:** Styliani Verykokou, Charalabos Ioannidis, Christos Angelopoulos

**Affiliations:** 1 Laboratory of Photogrammetry, School of Rural, Surveying and Geoinformatics Engineering, National Technical University of Athens, 15780 Athens, Greece; cioannid@survey.ntua.gr; 2 Department of Oral Diagnosis and Radiology, School of Dentistry, National and Kapodistrian University of Athens, 11527 Athens, Greece; angelopoulosc@gmail.com

**Keywords:** scaffold, bone graft, periodontitis, periodontal regeneration, cone beam computed tomography (CBCT), regenerative medicine, dentistry, tissue engineering, 3D modeling, 3D printing

## Abstract

The purpose of this article is to define and implement a methodology for the 3D design of customized patient-specific scaffolds (bone grafts) for the regeneration of periodontal tissues. The prerequisite of the proposed workflow is the three-dimensional (3D) structure of the periodontal defect, i.e., the 3D model of the hard tissues (alveolar bone and teeth) around the periodontal damage, which is proposed to be generated via a segmentation and 3D editing methodology using cone beam computed tomography (CBCT) data. Two types of methodologies for 3D periodontal scaffold (graft) design are described: (i) The methodology of designing periodontal defect customized block grafts and (ii) the methodology of designing extraction socket preservation customized grafts. The application of the proposed methodology for the generation of a 3D model of the hard tissues around periodontal defects of a patient using a CBCT scan and the 3D design of the two aforementioned types of scaffolds for personalized periodontal regenerative treatment shows promising results. The outputs of this work will be used as the basis for the 3D printing of bioabsorbable scaffolds of personalized treatment against periodontitis, which will simultaneously be used as sustained-release drug carriers.

## 1. Introduction

The possibilities of 3D printing in the fields of regenerative medicine and tissue engineering are immense [1]. There are numerous benefits to generating 3D-printed scaffolds, with one of the fundamental advantages being the possibility to model complex geometries for personalized regenerative treatment. In the last fifteen years, the progress in image-based and scanner-based 3D modeling technologies [2], such as computed tomography (CT) and cone beam computed tomography (CBCT), has significantly aided the development of customized scaffolds (bone grafts) using patient-specific anatomical data for medical applications as regards bone regeneration. Significant research has been conducted concerning 3D-printed scaffolds and related materials for tissue engineering applications [3,4,5,6,7,8,9,10,11,12]. So far, 3D printing techniques have been proposed for a wide variety of medical applications, ranging from bone tissue regeneration to tumor therapy, leading to the fabrication of scaffolds with controlled chemistry, interconnected porosity, and complex shapes [13].

In dentistry, 3D printing offers a multitude of advantages and has potential applications in various fields, including prosthodontics, oral and maxillofacial surgery, oral implantology, orthodontics, endodontics, and periodontology [14,15]. Treating patients with alveolar bone defects and restoring the bone structure is still a complex issue in clinical practice. In recent years, several applications have been reported using 3D-printed scaffolds for dental tissue engineering, including periodontal and alveolar bone regeneration [16,17,18,19,20,21,22,23,24,25,26,27,28,29,30,31,32,33,34,35,36,37]. Research has also been carried out on the use of different materials for the preparation of surgical guides for implant placement [38,39].

Similar to the aforementioned dental applications of 3D printing, the great majority of 3D-printed scaffolds for periodontal regeneration have not been designed using patient-specific data. Whereas the use of the CBCT technology has been suggested in the literature for the diagnosis of periodontal diseases [40] as CBCT datasets particularly assist in the accurate determination of the 3D morphology of periodontal defects, compared to 2D X-rays [41,42,43,44], the CBCT technology has not been sufficiently used for designing customized periodontal scaffolds. The first clinical treatment of periodontitis in a patient using a 3D-printed scaffold designed using CBCT data was performed in 2015 by Rasperini et al. [45]. Favorable results were obtained up to 12 months after the scaffold was placed on the patient. The generation of scaffolds using CBCT data has also been proposed in [46,47,48] for the reconstruction of alveolar bone defects. However, the research conducted so far on the 3D design of periodontitis scaffolds using CBCT data is very limited.

This article aims to fill the gap in the state-of-the-art research concerning the use of CBCT data for designing customized patient-specific 3D scaffolds (bone grafts) that can be 3D-printed for periodontal regeneration. In this context, it aims to determine and evaluate an efficient workflow for 3D modeling the hard tissues around the periodontal defect and designing 3D scaffolds based on the 3D morphology of the periodontal defect. The ultimate goal of this research is the creation of bioabsorbable 3D-printed scaffolds of personalized treatment against periodontitis, which will simultaneously be used as sustained-release drug carriers. In our previous work [49], we proposed an optimal methodology for 3D modeling of the alveolar bone and the teeth around periodontal defects using CBCT data, which was determined based on several experiments. In this article, we rely on our previous work by using and evaluating the proposed method for creating a 3D model of the teeth and the part of the alveolar bone where the periodontal defect is located. This 3D model is the prerequisite for the application of the methodology for designing personalized 3D periodontitis scaffolds, which is the theme of this article.

This article is structured as follows. Section 2 includes the materials and methods of our research. In Section 2.1, the workflow for creating 3D models of periodontal defects proposed in our previous work [49] is briefly summarized for the sake of completeness. Two types of methodologies for 3D periodontal scaffold design are described. The first type refers to the methodology of designing periodontal defect customized block grafts and is presented in Section 2.2. The second kind refers to the methodology of designing extraction socket preservation customized grafts and is presented in Section 2.3. Section 3 presents the results of applying the proposed methodology for the generation of a 3D model of the periodontal defects of a patient using CBCT data (Section 3.1) and the 3D design of the two types of scaffolds for personalized periodontal regeneration (Section 3.2). A discussion follows (Section 4).

## 2. Materials and Methods

In this section, the proposed methodology for 3D periodontal scaffold design is outlined. The initial data used for testing this methodology were the anonymized DICOM files of the mandibular CBCT scan of a patient diagnosed with periodontal disease by his/her dentist. The NewTom VGi EVO scanner was used, with a voxel size of 150 μm and a field of view (FOV) of 100 mm × 100 mm. The 3D Slicer open-source medical image editing software, version 4.11.20210226 [50] was used for the segmentation of the CBCT dataset and the generation of the first version of the 3D models of the alveolar bone and the teeth. The Geomagic Wrap 2017 commercial software package (3D Systems, Rock Hill, SC, USA—now developed and supported by Artec3D, Luxemburg City, Luxemburg) [51] was used to process the two generated 3D models of the alveolar bone and teeth, creating the final merged 3D model of the periodontal defect and designing two types of scaffolds for the specific patient, i.e., a periodontal defect customized block graft and an extraction socket preservation customized graft.

### 2.1. 3D Modeling of Periodontal Defects

In our previous research [49], we evaluated segmentation and 3D modeling workflows using dental CBCT data by performing several comparisons between the 3D models of the hard tissues of the oral cavity of a patient with periodontitis, generated through different methodologies. This research led to the development of an optimal methodology that may be used for 3D modeling of the teeth and the alveolar bone of patients with periodontitis in the specific regions where the periodontal defects are observed. The detailed methodology was previously described by Verykokou et al. [49] and is summarized for the sake of completeness in the following:

1. Definition of the region of interest by cropping the corresponding volume defined in the CBCT dataset.
2. Definition of three segments: Teeth, alveolar bone, and “other”, including all the other areas of the oral system, depicted in the CBCT cropped images.
3. Definition of samples for the aforementioned segments for a small subset of CBCT images (e.g., 30 images: 10 per reference plane), through a combination of automatic (thresholding) and manual procedures.
4. Initialization of the “Grow from seeds” method [52].
5. Iterative correction and update of the result of the “Grow from seeds” method until the visual inspection of the result satisfies the user.
6. Conversion of the segmentation results for the teeth and alveolar bone segments into 3D models (meshes) in STL format.
7. Editing of the 3D models of teeth and alveolar bone, including, indicatively, the conversion of each 3D mesh to a point cloud; the editing of the point cloud; the creation of a 3D mesh (surface) using the edited point cloud; and the optional merging of the two 3D models into a single 3D model and storage in STL format.

As discussed in detail in [49], with the exception of the last step of the aforementioned methodology, which may be accomplished using a 3D editing software such as Geomagic Wrap (3D Systems, Rock Hill, SC, USA—now developed and supported by Artec3D, Luxemburg City, Luxemburg) [51], all the other steps may be implemented using medical imaging software such as the open-source 3D Slicer software solution [50].

### 2.2. Design of Periodontal Defect Customized Block Grafts

**Figure 1:**
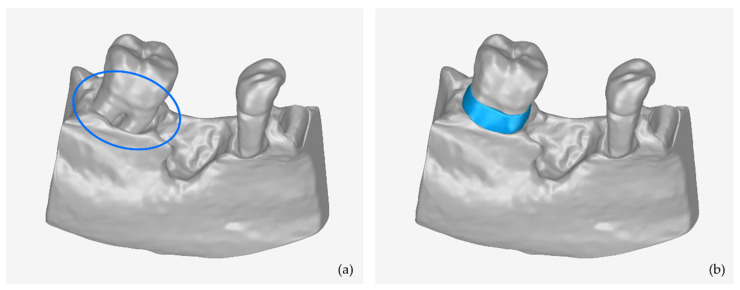
(**a**) Example of periodontal defect (marked with blue ellipse). (**b**) periodontal defect customized block graft (blue-color 3D model).

In this section, a methodology is presented for creating the 3D model of a periodontal defect customized block graft based on the existing 3D model of the part of the alveolar bone and the teeth where the periodontal defect is concentrated. This methodology was determined based on multiple tests using real data. A key feature of this type of scaffold is the fact that its inner surface is in contact with the root of the tooth and the alveolar bone, while its outer surface is the bony outline of the missing alveolar bone. This scaffold is designed to replace the lost bone around the tooth due to periodontitis (inflammation of the bone around the teeth). An example of a periodontal lesion and the corresponding scaffold is shown in Figure 1.

#### 2.2.1. Stage A: Design of an Initial Approximation of the Scaffold Internal Surface

The first stage of the process of designing the 3D model of a periodontal defect customized block graft (stage A) is the creation of an initial approximation of the internal surface of the scaffold, i.e., the surface of the scaffold that touches the tooth (laterally) and the alveolar bone (at the bottom of the scaffold if it is a mandibular scaffold or on the upper part of the scaffold if it is a maxillary scaffold).

The initial step of stage A (stage A1) is the cutting of the 3D model of the teeth and the alveolar bone so that the final 3D model covers exactly the area of the scaffold. This model—after further processing in the subsequent steps of the process—will constitute the inner surface of the scaffold.

During the next step of stage A (stage A2), the 3D model produced in stage A1 undergoes optional processing, if it is necessary. Indicative processing steps that the model may need to undergo during stage A2 are hole filling, noise reduction, 3D surface smoothing, further cropping, etc.

The normal vectors of the above 3D surface (i.e., the result of stage A2) have the opposite direction, i.e., they are inverted, as this surface is part of the 3D model of the teeth and the alveolar bone. Thus, the normal vectors must be corrected to point in the correct direction. This process, i.e., the inversion of the normal vectors of the model, which is the result of step A2, is performed in the current step (step A3). The result of this step is the initial approximation of the inner surface of the scaffold, which now has correct normal vectors.

**Figure 2:**
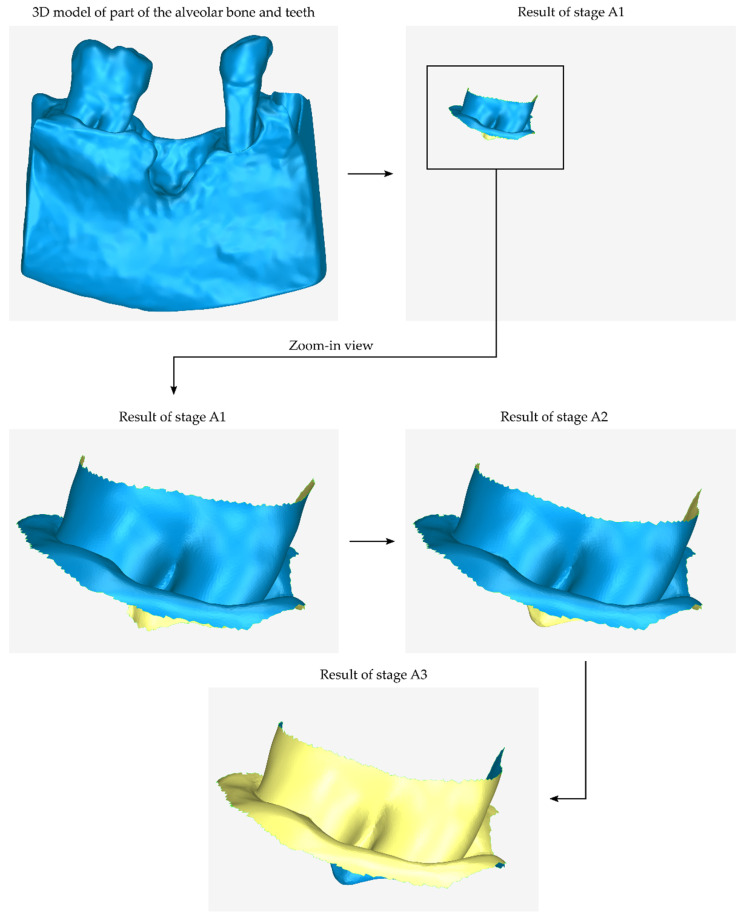
Graphical example of the procedure performed during stage A of the proposed periodontal defect customized block graft design methodology.

A graphical representation of the process performed during stage A (stages A1, A2, and A3) is shown in Figure 2. In this image, the surface with normal vectors outward (i.e., the outer side of the 3D model—the side that appears when the 3D model is printed) is represented in blue, while the inner side of the 3D model (i.e., the side not visible when the model is printed) is represented in yellow.

#### 2.2.2. Stage B: Design of the Scaffold Outer Surface

The next stage of the process of creating a 3D periodontal defect customized block graft (stage B) is the design of the external surface of the scaffold, i.e., the surface of the scaffold that does not touch the patient’s tooth and alveolar bone. During the first step of stage B (stage B1), the following procedures are performed:

- Copying the model that has been cut from the original 3D model of the part of the alveolar bone and the teeth in which the periodontal defect is concentrated (model that is the result of stage A1).
- Cutting the above model, so that its new surface covers approximately the area that will be occupied by the outer surface of the scaffold.
- Moving the above model by the intended distance according to the desired average thickness of the scaffold (e.g., 1 mm).
- Processing the above model (e.g., further cutting, filling gaps, etc.).
- The model that is the final result of stage B1—after further processing in subsequent steps of stage B—will constitute the outer surface of the scaffold.

**Figure 3:**
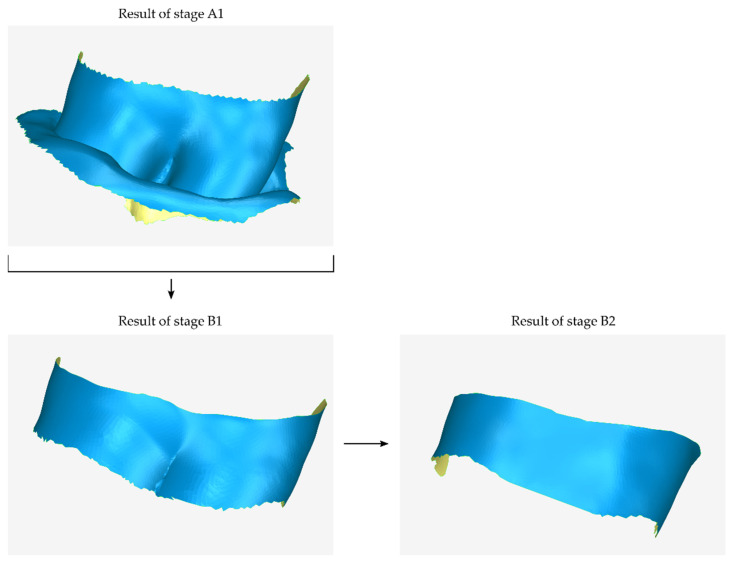
Graphical example of the procedure performed during stages B1 and B2 of the proposed periodontal defect customized block graft design methodology.

During the next step of stage B (stage B2), the 3D model of the first approximation of the outer surface of the scaffold, i.e., the result of stage B1, undergoes a rotation transformation and—possibly—translation and scaling, so that it has the desired slope, forming—finally—a scaffold of variable thickness (i.e., of greater thickness near the surface of the alveolar bone and less thickness in the opposite direction (upwards if it is a mandibular scaffold or downwards if it is a maxillary scaffold). Moreover, at this stage, the model undergoes further processing, which may include smoothing, noise reduction, erasing of part of the surface and filling of the said hole with a new surface of different curvature, and so on. The result of this process is the second approximation of the outer surface of the scaffold. A graphic representation of the first two steps of stage B (stages B1–B2) is shown in Figure 3.

**Figure 4:**
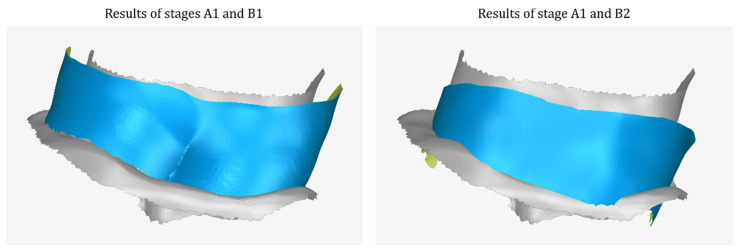
Example results of stage A1 (gray) and stage B1 (blue) (**left**) as well as stage A1 (gray) and stage B2 (blue) (**right**) in the same 3D space so that the geometric transformations and the processing that the 3D model of stage A1 underwent during stages B1 and B2 are visualized.

Figure 4 shows examples of the results of stage A1 and stage B1, as well as stages A1 and B2 in the same 3D space, so that the translation and processing that the 3D model of stage A1 underwent during stage B1, as well as the translation—rotation—scaling transformation and its further processing, during stage B2, are visualized.

From the 3D model of the outer surface of the scaffold created during stage B2, it is possible that parts of its lateral surfaces (i.e., the surfaces located at the junction of the inner and outer surfaces of the scaffold) are missing. In this case—and if these surfaces cannot result from a gap-filling process after merging the inner with the outer surface—they need to be designed at this stage. Thus, during the third and fourth steps of stage B (steps B3 and B4), the lateral parts of the outer surface of the scaffold that may be missing from the 3D model of stage B2 are designed. Of course, in the case that there is no lateral part of the outer surface of the model missing, these steps are omitted.

In the context of stage B3, for each lateral surface that needs to be created, the following procedures are performed:

- Copying of the model that has undergone a cutting from the original 3D model of the periodontal defect (model that is the result of stage A1).
- Cutting of the above model, so that its new surface covers approximately the area that will be occupied by the lateral outer surface of the scaffold that is missing from the previous outer surface (i.e., from the result of stage B2) and is approximately parallel to the latter.
- Translation of the above model by the intended distance according to the desired average thickness of the scaffold.

The result of stage B3 is the first approximation of each lateral surface that is missing from the model of stage B2. However, taking into account the fact that the scaffold has to be of varying thickness (greater thickness near the surface of the alveolar bone and smaller thickness in the opposite direction), any lateral surface, created by translating part of the tooth, needs to be rotated, in order to have the desired inclination and—if necessary—undergo an optional rescaling and optional further processing (e.g., smoothing). These processes are performed in the context of stage B4. The result of the process performed in stage B4 is the final approximation of the lateral surfaces that are missing from the outer surface of the scaffold created in stage B2.

**Figure 5:**
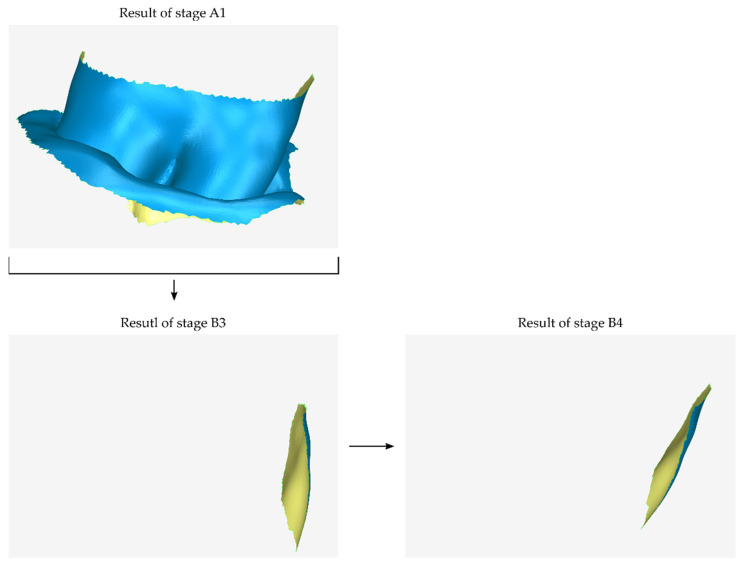
Graphical example of the procedure performed during stages B3 and B4 of the proposed periodontal defect customized block graft design methodology.

A graphical representation of stages B3 and B4 is shown in Figure 5. In the example case illustrated in the images of this article, one lateral surface is missing from the model of the outer surface of the scaffold (stage B2). The generated model was translated relative to the 3D model of the cropped tooth (stage B3) and rotated (stage B4) without scaling or further processing.

The merging of the models of the lateral surface(s) produced during stage B4 with the model of the outer surface of stage B2 follows. This process is performed within stage B5.

During step B6, the 3D model of step B5 undergoes further processing, which consists of cutting parts of the surface, filling the resulting holes, and smoothing the parts resulting from hole filling.

**Figure 6:**
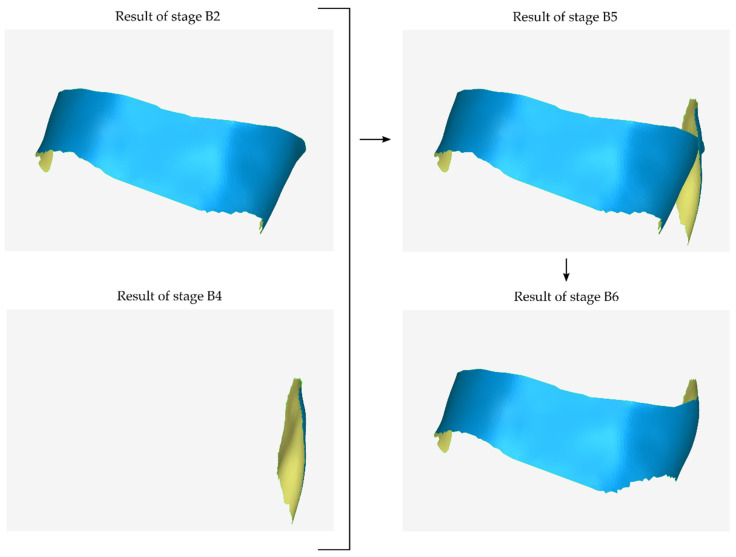
Graphical example of the process performed during steps B5 and B6 of the proposed periodontal defect customized block graft design methodology.

Stages B5 and B6 are applied as long as stages B3 and B4 are preceded. A graphical representation of stages B5 and B6 is shown in Figure 6.

During the next step of stage B (stage B7), the surface of the part of the alveolar bone on which the scaffold will lay (result of stage A3) is used to limit the outer surface of the scaffold (result of stage B6), which is further cut.

The next step of stage B (stage B8) is the—optional—design of part of the upper outer surface of the scaffold (if it is a mandibular scaffold) or of the lower outer surface of the scaffold (if it is a maxillary scaffold), in the case where part of it is missing (that is, if the result of stage B7—or more generally of the last stage that has been implemented—has a lower height than the intended one in part of the surface). The creation of the missing upper or lower missing part of the surface may be carried out by following the steps described below:

- Duplication of the model, which is the last approximation of the external surface of the scaffold (the result of stage B7).
- Cutting of the above model, so that its surface covers approximately the upper part of the missing surface.
- Translation of the above model by the intended distance upwards (to cover the upper part of the scaffold surface, if it is a mandibular scaffold) or downwards (to cover the lower part of the scaffold surface, if it is a maxillary scaffold).
- Merging of the above model with the model that is the last approximation of the outer surface of the scaffold (the result of stage B7).
- Processing of the merged model, which may include gap filling, smoothing, etc.

**Figure 7:**
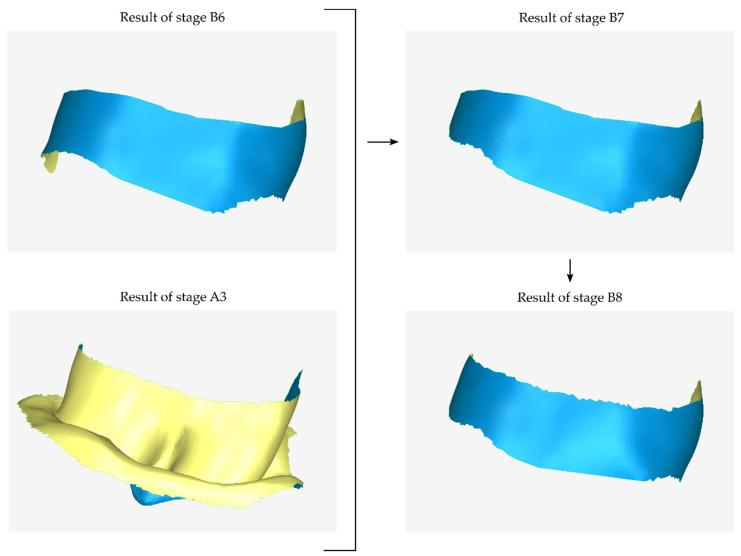
Graphical example of the procedure performed during steps B7 and B8 of the proposed periodontal defect customized block graft design methodology.

Additionally, this stage, after creating the missing surfaces, may include further trimming of the 3D model so that its surface forms the final outer surface of the scaffold. Stage B8 is applied in the case where the 3D model of the external surface of the scaffold needs it (i.e., for a surface of a lower height than the desired one). A graphical representation of stages B7 and B8 is shown in Figure 7. In this specific case of the example, from the result of stage B7, part of the upper left (as the reader “sees” in Figure 7) surface of the scaffold is “missing”. This part was created during stage B8, at the end of which further cutting was applied to the 3D model.

#### 2.2.3. Stage C: Design of a Final Approximation of the Scaffold Internal Surface

**Figure 8:**
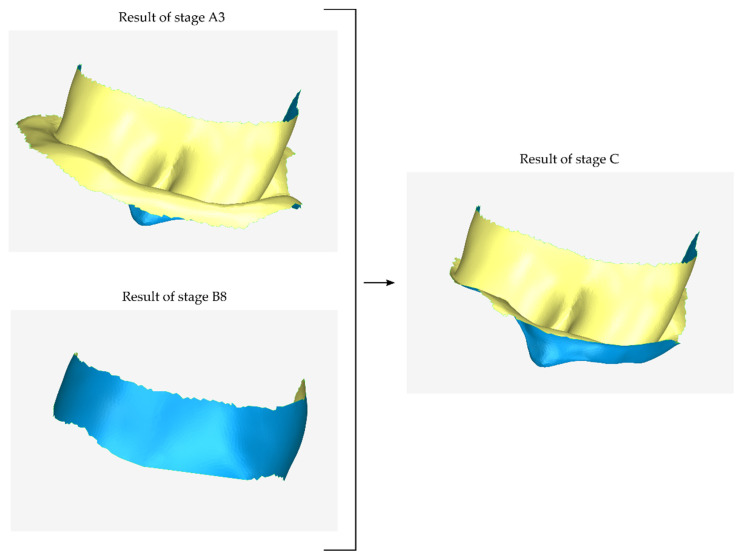
Graphical example of the procedure performed during stage C of the proposed periodontal defect customized block graft design methodology.

During the next stage (stage C), the model produced in stage B (the outer surface of the scaffold) is used to further trim the model produced in stage A (initial approximation of the inner surface of the scaffold). The result of this process is the final approximation of the inner surface of the scaffold. A graphical illustration of the process performed during stage C is shown in Figure 8.

#### 2.2.4. Stage D: Design of the Final Scaffold

During the first step of the final stage of scaffold design (stage D1), the external (stage B) and internal (stage C) surfaces of the 3D model of the scaffold are merged. The result of this process is the first approximation of the 3D model of the scaffold.

**Figure 9:**
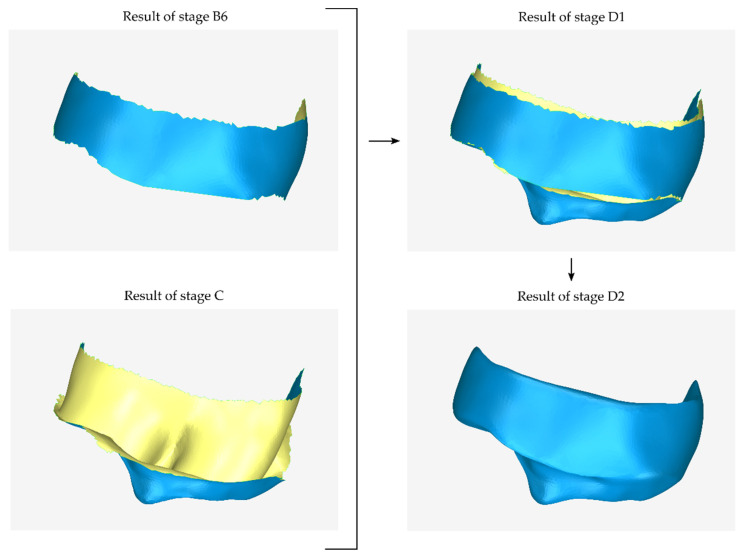
Graphical example of the procedure performed during stage D of the proposed periodontal defect customized block graft methodology.

The last step of the periodontal defect customized block graft design process (stage D2) is the creation of the surfaces that are missing from the scaffold, i.e., the upper surface (in the case of a mandibular scaffold) or the lower surface (in the case of a maxillary scaffold) and the lateral surfaces of the scaffold, as well as its processing. This is a process of step-by-step hole filling and final processing of the 3D model. Specifically, the result of stage D1 is an open surface consisting of the inner surface of the scaffold—that is, the surface that is in contact with the patient’s tooth and alveolar bone—and its outer surface. The surfaces joining the inner and outer sides of the scaffold (two side surfaces and the top or bottom surface) are created during stage D2 through the hole-filling processes. After designing the closed surface of the scaffold, the 3D scaffold is further processed (e.g., by erasing surface parts with undesired curvature and filling holes, smoothing, etc.) so that it comes to a final form. A graphical illustration of the process performed during stage D is shown in Figure 9.

### 2.3. Design of an Extraction Socket Preservation Customized Graft

**Figure 10:**
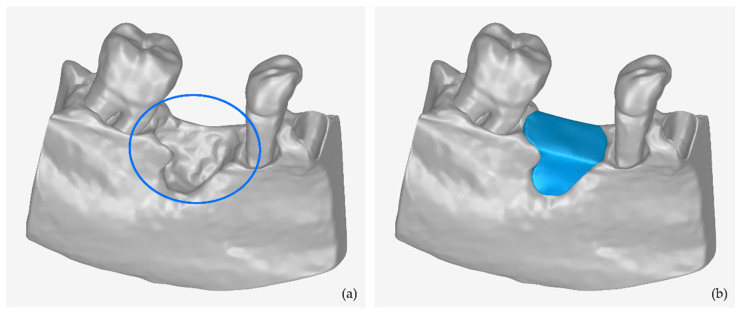
(**a**) Example of periodontal defect (marked with blue ellipse). (**b**) Extraction socket preservation customized graft (blue-color 3D model).

**Figure 11:**
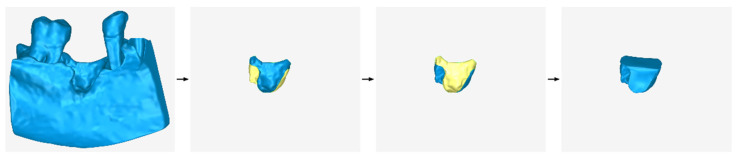
Graphical example of the methodology of designing an extraction socket preservation customized graft.

A key feature of this type of scaffold is that it is the complement to the bone loss (hole) that results from the extraction of a tooth. The peripheral-external surfaces of the scaffold are in contact with the inner surface of the hole and its free surface (upper surface if it is a mandibular scaffold or lower surface if it is a maxillary scaffold) follows the contour of the bone in the specific anatomical area. The new surfaces to be created can be treated as hole filling. In this case, some of the stages of the methodology described in Section 2.2 are omitted, because its external surface is not designed from the beginning, as is the case for the general methodology of designing a periodontal defect customized block graft, described in Section 2.2. An example of a periodontal defect and the corresponding extraction socket preservation customized graft filling the alveolar bone cavity is shown in Figure 10. The proposed stages are described below (in the sections that follow) and are graphically illustrated in Figure 11.

#### 2.3.1. Stage A: Design of the Scaffold Internal Surface

This stage is equivalent to stage A of the periodontal defect customized block graft design methodology. For the sake of completeness, it is summarized below.

In the first step of stage A (stage A1), the 3D model of the teeth and alveolar bone is cut so that the final 3D model covers exactly the area of the scaffold. This model will constitute—after further processing in subsequent steps of stage A—the internal surface of the scaffold, i.e., the surface of the scaffold that is in contact with the tooth and the alveolar bone. This stage is analogous to stage A1 of the periodontal defect customized block graft design methodology.

In a subsequent step of stage A (stage A2), an optional (if necessary) processing of the above 3D model is carried out. Indicative processing steps that this model may need to undergo are hole filling, noise reduction, 3D surface smoothing, further cropping, etc. This stage is analogous to stage A2 of the periodontal defect customized block graft design methodology. This step did not need to be applied in the example shown in Figure 11.

The normal vectors of the above model have the opposite direction, as this surface is part of the 3D model of the alveolar bone. Thus, the inverted normal vectors must be corrected to point in the correct direction. This process, i.e., the inversion of the normal vectors of the model that is the result of step A2 (if it has been applied) or step A1 (if step A2 has been omitted), is performed in step A3. The result of this process is the final approximation of the inner surface of the scaffold, which now has correct normal vectors. This stage is analogous to stage A3 of the periodontal defect customized block graft design methodology.

#### 2.3.2. Stage B: Design of Final Scaffold

In the final stage (stage B) of the methodology of designing an extraction socket preservation customized graft, the missing surfaces of the scaffold are created, i.e., the upper surface (in the case of a mandibular scaffold) or the lower surface (in the case of a maxillary scaffold), as well as the external surface(s) of the scaffold, and the latter is processed so that it has its final form.

The design of the aforementioned surfaces may be conducted through a hole-filling methodology, which may involve dividing the hole into smaller holes by designing a “bridge”, i.e., a surface joining two ends of the hole. After the hole-filling procedure, the final processing of the model follows, which may include erasing parts of the surface with undesired curvature and filling holes, noise reduction, smoothing, etc. This stage is analogous to stage D of the periodontal defect customized block graft design methodology.

**Figure 12:**
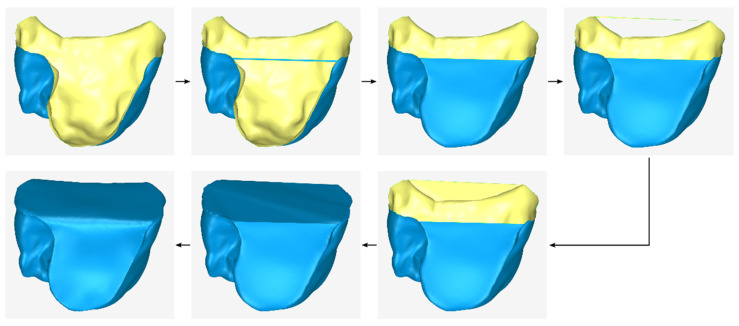
Graphical example of the process performed during stage B of the proposed extraction socket preservation customized graft design methodology.

A graphical example of hole filling for designing the upper surface and the two lateral surfaces of a mandibular extraction socket preservation customized graft and its processing is illustrated in Figure 12.

Similar to the case of a periodontal defect customized block graft, the designed extraction socket preservation customized graft is recommended to be exported into STL format, optimized for 3D printing. Furthermore, specifically for the case of an extraction socket preservation customized graft, it is recommended, before saving the 3D model of the scaffold, to scale it down by a factor of 0.98, so as to ensure that the 3D model of the scaffold fits the jaw of the patient. The reason for the proposed rescaling of the model lies in the fact that the inner surface of the scaffold is the surface of the part of the alveolar bone where the periodontal lesion is located. Therefore, this small reduction in the scale of the 3D model of the scaffold ensures that it can be better applied to the patient’s jaw.

## 3. Results

### 3.1. 3D Model of Hard Tissues in the Area of the Periodontal Defects

The 3D modeling methodology introduced in [49] and summarized in Section 2.1 was followed to create a 3D model of the alveolar bone and the teeth for part of the mandible of a patient with periodontitis. This model is used as a basis for the 3D design of periodontal scaffolds.

**Figure 13:**
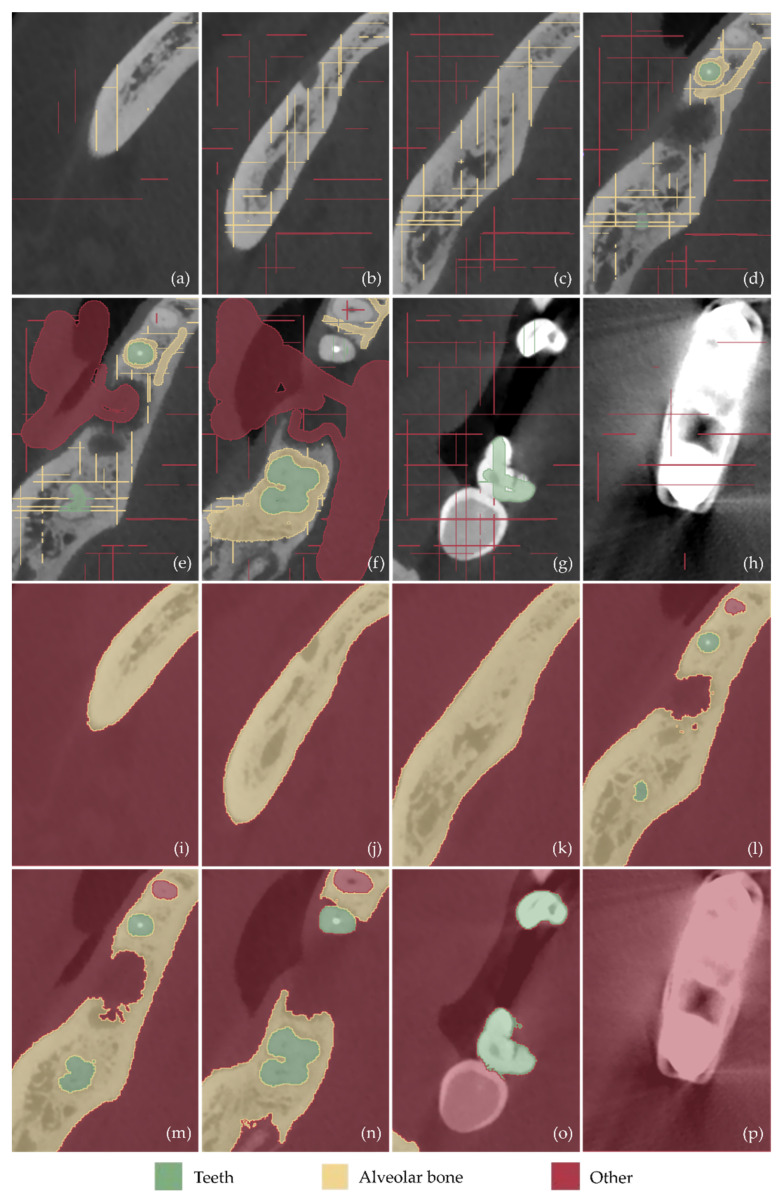
(**a**–**h**) Samples manually defined for the segments teeth, alveolar bone, and “other” in a subset of the tomographic images (after their cropping, in order to depict the region of interest) in the axial plane. (**i**–**p**) Segmentation results using the “Grow from Seeds” method for the tomographic images (**a**–**h**). Specifically, sub-figure (**i**) shows the result of the “Grow from seeds” method for the tomographic image shown in (**a**) z sub-figure (**j**) shows the result of the “Grow from seeds” method for the tomographic image shown in (**b**); sub-figure (**k**) shows the result of the “Grow from seeds” method for the tomographic image shown in (**c**); sub-figure (**l**) shows the result of the “Grow from seeds” method for the tomographic image shown in (**d**); sub-figure (**m**) shows the result of the “Grow from seeds” method for the tomographic image shown in (**e**); sub-figure (**n**) shows the result of the “Grow from seeds” method for the tomographic image shown in (**f**); sub-figure (**o**) shows the result of the “Grow from seeds” method for the tomographic image shown in (**g**); and sub-figure (**p**) shows the result of the “Grow from seeds” method for the tomographic image shown in (**h**).

**Figure 14:**
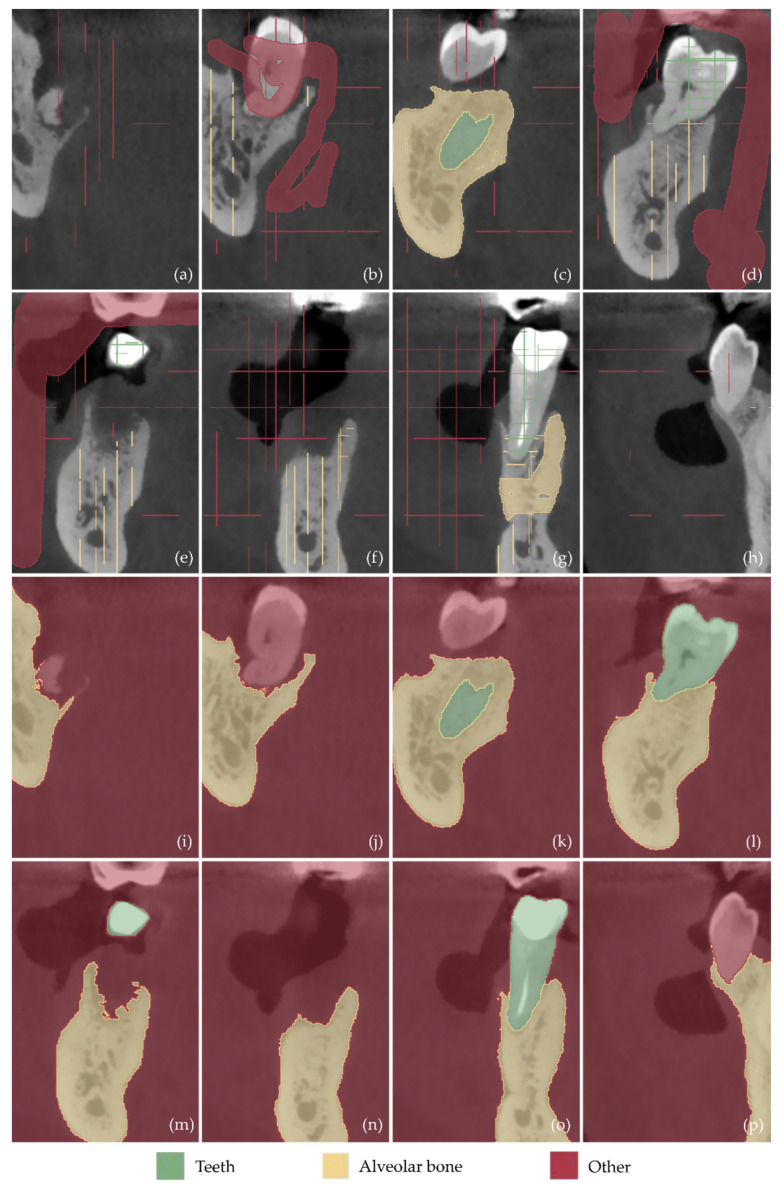
(**a**–**h**) Samples manually defined for the segments teeth, alveolar bone, and other in a subset of the tomographic images (after their cropping, in order to depict the region of interest) in the coronal plane. (**i**–**p**) Segmentation results using the “Grow from Seeds” method for the tomographic images (**a**–**h**). Specifically, sub-figure (**i**) shows the result of the “Grow from seeds” method for the tomographic image shown in (**a**); sub-figure (**j**) shows the result of the “Grow from seeds” method for the tomographic image shown in (**b**); sub-figure (**k**) shows the result of the “Grow from seeds” method for the tomographic image shown in (**c**); sub-figure (**l**) shows the result of the “Grow from seeds” method for the tomographic image shown in (**d**); sub-figure (**m**) shows the result of the “Grow from seeds” method for the tomographic image shown in (**e**); sub-figure (**n**) shows the result of the “Grow from seeds” method for the tomographic image shown in (**f**); sub-figure (**o**) shows the result of the “Grow from seeds” method for the tomographic image shown in (**g**); and sub-figure (**p**) shows the result of the “Grow from seeds” method for the tomographic image shown in (**h**).

**Figure 15:**
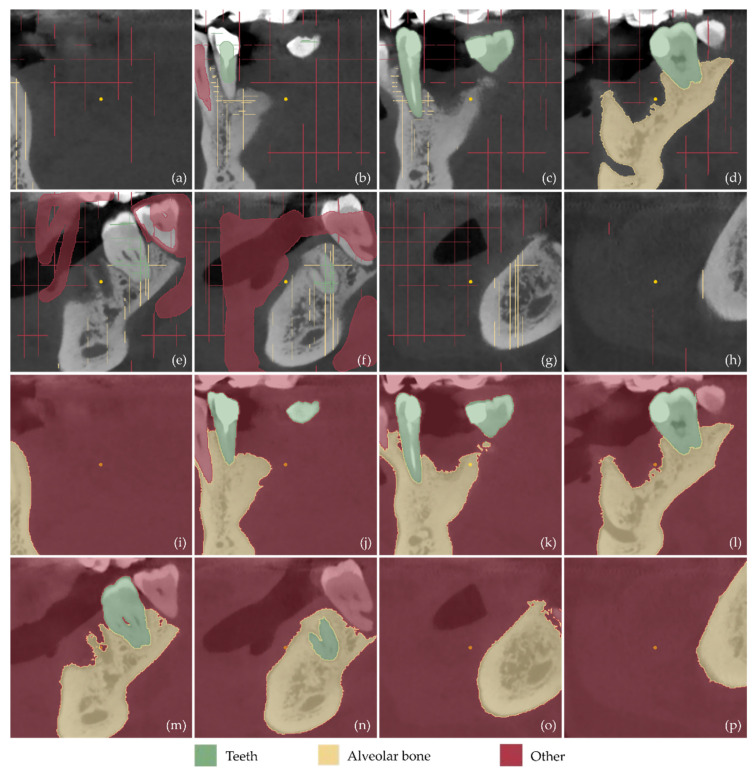
(**a**–**h**): Samples manually defined for the segments teeth, alveolar bone, and other in a subset of the tomographic images (after their cropping, in order to depict the region of interest) in the sagittal plane. (**i**–**p**): Segmentation results using the “Grow from Seeds” method for the tomographic images (**a**–**h**). Specifically, sub-figure (**i**) shows the result of the “Grow from seeds” method for the tomographic image shown in (**a**); sub-figure (**j**) shows the result of the “Grow from seeds” method for the tomographic image shown in (**b**); sub-figure (**k**) shows the result of the “Grow from seeds” method for the tomographic image shown in (**c**); sub-figure (**l**) shows the result of the “Grow from seeds” method for the tomographic image shown in (**d**); sub-figure (**m**) shows the result of the “Grow from seeds” method for the tomographic image shown in (**e**); sub-figure (**n**) shows the result of the “Grow from seeds” method for the tomographic image shown in (**f**); sub-figure (**o**) shows the result of the “Grow from seeds” method for the tomographic image shown in (**g**); and sub-figure (**p**) shows the result of the “Grow from seeds” method for the tomographic image shown in (**h**).

Examples of samples manually defined in a subset of the CBCT images (after cropping them to include only the region of interest) are shown in Figure 13a–h (tomographic images in the axial plane), Figure 14a–h (tomographic images in the coronal plane), and Figure 15a–h (tomographic images in the sagittal plane). These images show the result of the first three steps of the methodology described in Section 2.1, namely the cutting of the volume of interest (step 1), the definition of the segments teeth, alveolar bone, and “other” (step 2), and the definition of samples for these segments for a small subset of tomographic images (step 3).

The result of the next two steps of the 3D modeling methodology, i.e., the initialization of the “Grow from seeds” method (step 4) and the iterative correction and update of the results of the “Grow from seeds” method (step 5), is shown in Figure 13i–p for the axial plane, in Figure 14i–p for the coronal plane, and in Figure 15i–p for the sagittal plane. From the aforementioned figures (Figure 13, Figure 14 and Figure 15), it is easy to see the result of the “Grow from seeds” method (sub-figures (i–p)) based on the definition of the samples depicted in the same tomographic images (sub-figures (a–h)).

From the sub-figures (i–p) of Figure 13, Figure 14 and Figure 15, it becomes clear that the “Grow from seeds” method yields satisfactory segmentation results. During step 5 of the visual inspection of the result of the “Grow from seeds” method and iterative correction and update of the samples, the errors that are deemed to be easily corrected by processing the generated 3D model have not been corrected. For example, in Figure 13l, the small areas within the alveolar bone that were characterized as “other” via the “Grow from seeds” method should also belong to the alveolar bone segment. However, it was found that such failures are easier and/or faster to correct using 3D model editing software. Similarly, noise is included in the boundaries of the alveolar bone in the axial plane in Figure 13m,n and in the teeth boundaries at the axial plane in Figure 13o. Noise removal and boundary smoothing are similarly implemented in a faster way using a 3D model processing software than their correction in—more than one—tomographic images in the three reference planes. Similar failures in the estimated boundaries of segments via the “Grow from seeds” method, primarily due to the existence of noise, are also evident in Figure 14i–p (coronal plane) and Figure 15i–p (sagittal plane).

**Figure 16:**
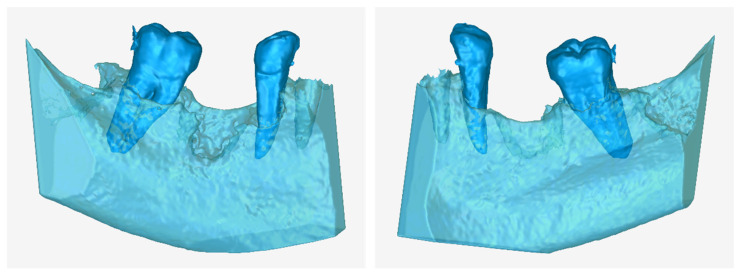
3D models of teeth and alveolar bone before processing, as obtained from the segmentation process, from two different viewpoints. The alveolar bone model is rendered transparent so that the parts of the teeth inside it are visible.

Figure 16 shows—in the same 3D space, from two different viewing points—the 3D models of the teeth and the alveolar bone, as they resulted from the segmentation process, before any processing. This is the result of step 6 of the 3D modeling methodology discussed in Section 2.1, during which the 3D models are saved in STL format.

**Figure 17:**
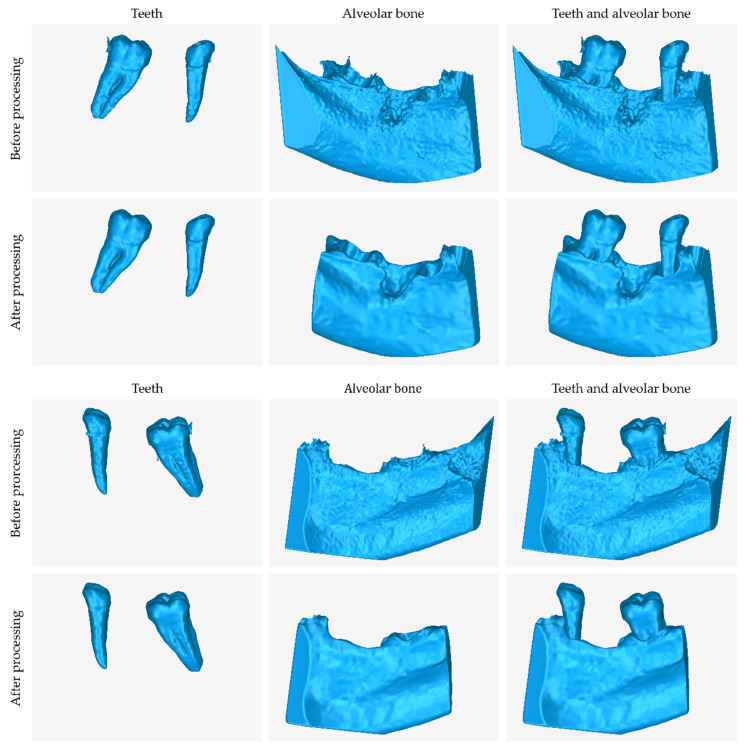
3D models of teeth (**left**), alveolar bone (**center**), and teeth and alveolar bone (**right**) before and after processing, from two different viewing points.

In Figure 17, the following 3D models of hard tissues around the periodontal defect are depicted from two different viewing angles, before their processing (the result of step 6 of the proposed 3D modeling methodology) and after their processing (the result of step 7 of the proposed 3D modeling methodology):

- The 3D teeth model.
- The 3D alveolar bone model.
- The 3D model of teeth and alveolar bone.

From the said image, the difference between the 3D model resulting from the segmentation process and the final 3D model, after its processing, is noticeable; hence, its processing in a 3D model editing software is deemed necessary.

**Figure 18:**
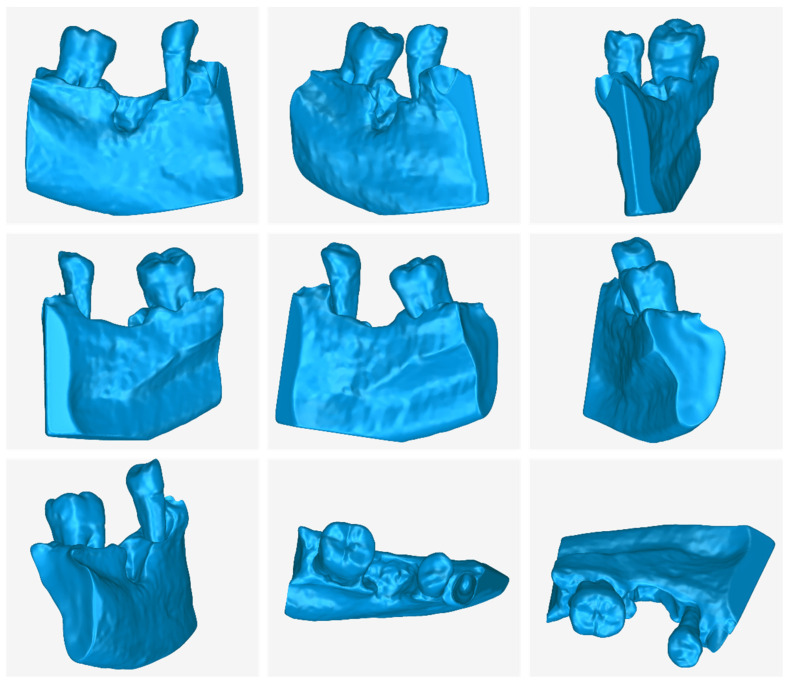
Final 3D model of teeth and alveolar bone (after processing) from nine different viewpoints.

**Figure 19:**
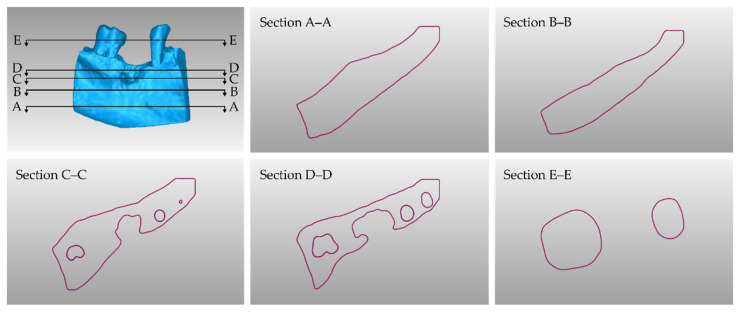
Sectional drawings (sections A–A; B–B; C–C; D–D; and E–E) of the final 3D model of teeth and alveolar bone using five different section planes (shown in the upper left sub-figure).

Table 1 shows the number of triangles and the size of the 3D models of teeth (before processing) and alveolar bone (before processing), as well as the final merged teeth and alveolar bone 3D model (after its processing). The final 3D model of teeth and alveolar bone (after processing) is shown in Figure 18. Finally, Figure 19 shows sectional drawings (sections) of the final 3D model of teeth and alveolar bone (after its processing) using five different section planes.

### 3.2. 3D Models of Scaffolds

The two scaffolds used in the example images of Section 2.2 and Section 2.3 to graphically illustrate the 3D periodontal scaffold design process refer to the same patient and are presented in this section. The 3D model of the part of the alveolar bone and the teeth where the periodontal defects of the particular patient are located (Section 3.1) was used as initial data for designing the two scaffolds.

**Figure 20:**
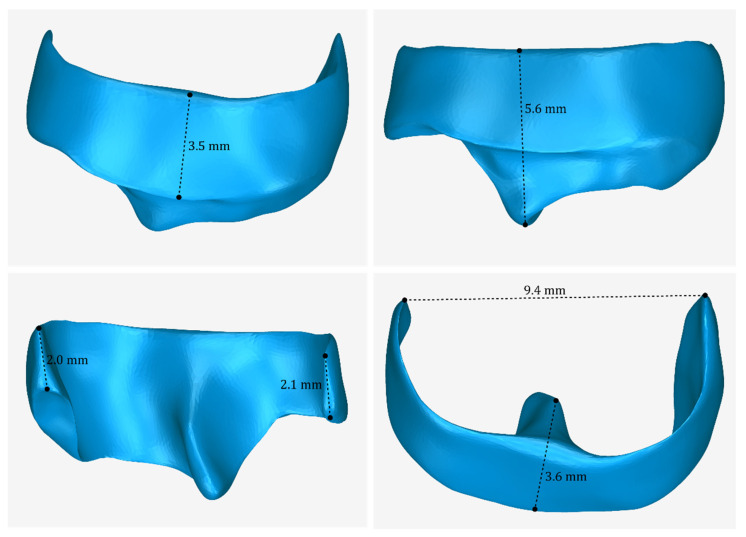
3D model of the periodontal defect customized block graft from different viewpoints and illustration of selected dimensions thereof.

**Figure 21:**
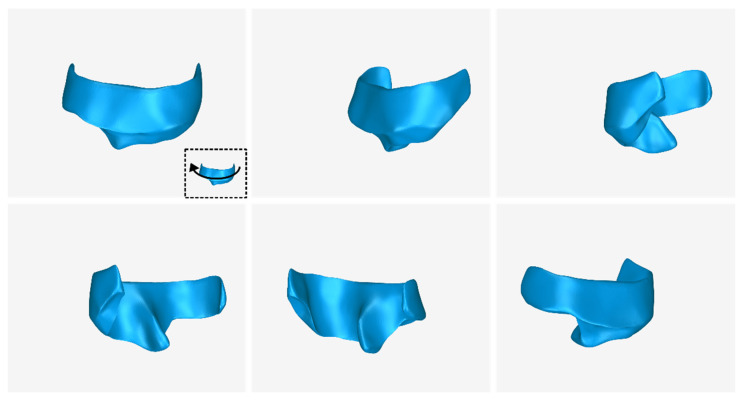
3D model of the periodontal defect customized block graft from different viewpoints, resulting from the rotation of the 3D model to the left.

**Figure 22:**
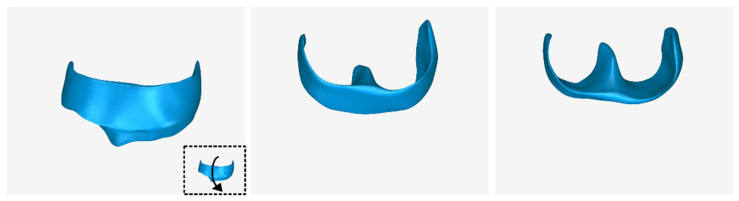
3D model of the periodontal defect customized block graft from different viewpoints, resulting from the rotation of the 3D model downwards.

**Figure 23:**
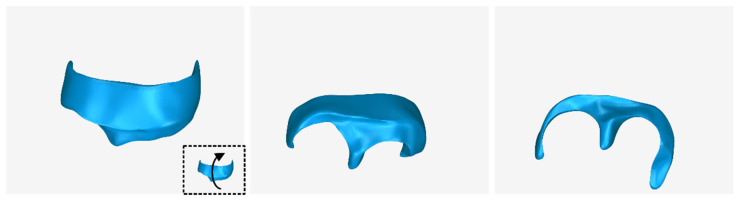
3D model of the periodontal defect customized block graft from different viewpoints, resulting from the rotation of the 3D model upwards.

**Figure 24:**
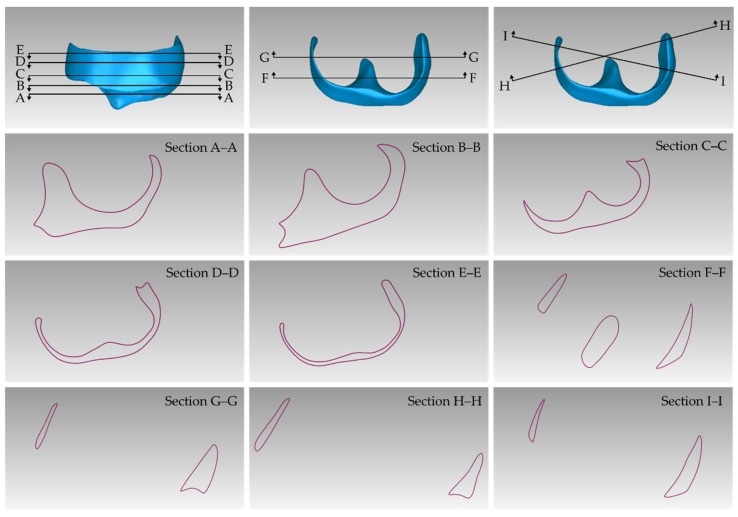
Sectional drawings (sections A–A; B–B; C–C; D–D; E–E; F–F; G–G; H–H; and I–I) of the periodontal defect customized block graft using nine different section planes (illustrated in the top three sub-figures).

The 3D model of the periodontal defect customized block graft, which was designed according to the methodology mentioned in Section 2.2, as well as its dimensions, is shown in Figure 20. Its volume is 52.18 mm^3^. It consists of 15,560 triangles and has a size of 0.8 MB. Snapshots of this 3D model from different viewpoints are listed in Figure 21, Figure 22 and Figure 23. Finally, Figure 24 shows sectional drawings of the 3D model of the graft using nine different section planes.

**Figure 25:**
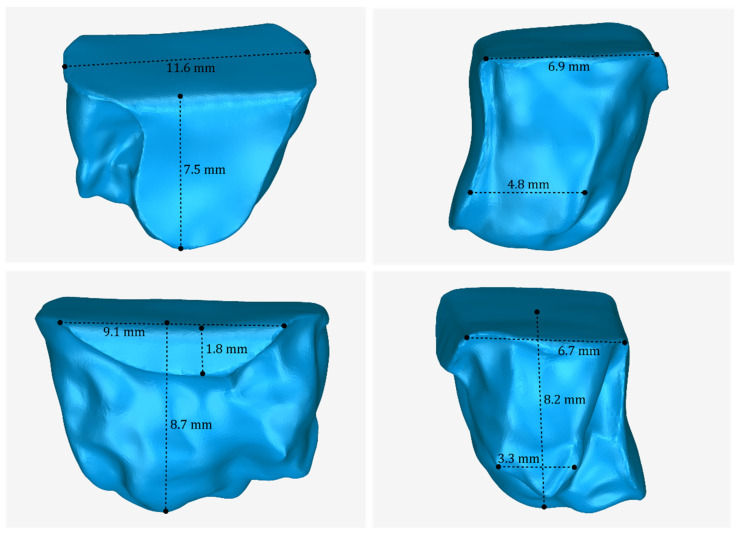
3D model of the extraction socket preservation customized graft from different viewpoints and illustration of selected dimensions thereof.

**Figure 26:**
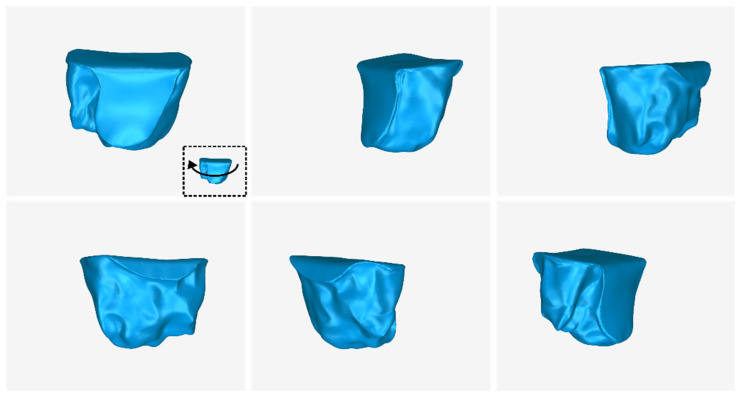
3D model of the extraction socket preservation customized graft from different viewpoints, resulting from the rotation of the 3D model to the left.

**Figure 27:**
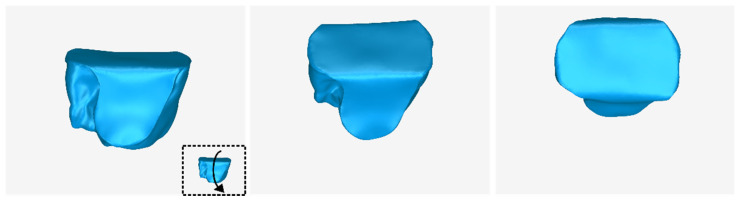
3D model of the extraction socket preservation customized graft from different viewpoints, resulting from the rotation of the 3D model downwards.

**Figure 28:**
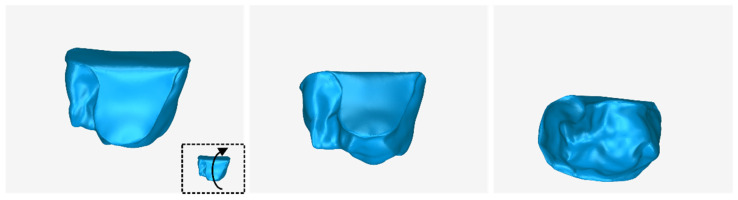
3D model of the extraction socket preservation customized graft from different viewpoints, resulting from the rotation of the 3D model upwards.

**Figure 29:**
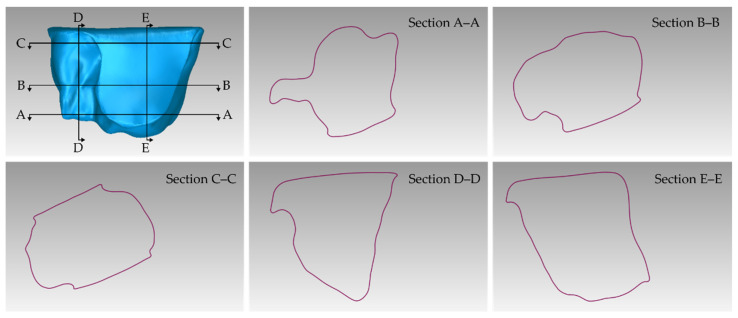
Sectional drawings (sections A–A; B–B; C–C; D–D; and E–E) of the extraction socket preservation customized graft using five different section planes (which are shown in the upper left sub-figure).

The 3D model of the extraction socket preservation customized graft, which was designed according to the methodology mentioned in Section 2.3, as well as its dimensions, is shown in Figure 25. Its volume is 418.08 mm^3^. It consists of 36,728 triangles and has a size of 1.8 MB. Snapshots of this 3D model from different viewpoints are illustrated in Figure 26, Figure 27 and Figure 28. Finally, Figure 29 shows sectional drawings of the 3D model of the scaffold using five different section planes.

**Figure 30:**
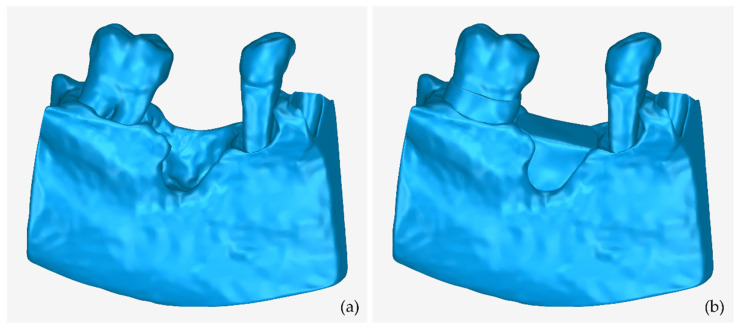
(**a**) 3D model of the part of the alveolar bone and teeth where the periodontal lesions are located. (**b**) 3D model of the part of the alveolar bone and teeth where the periodontal lesions are located and 3D models of scaffolds.

**Figure 31:**
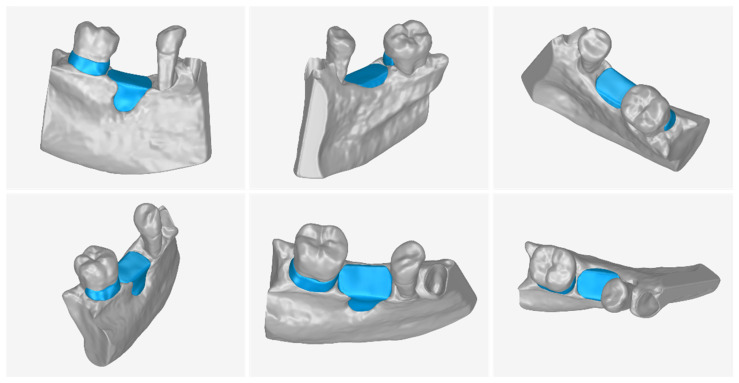
3D model of the part of the alveolar bone and teeth where the periodontal lesions are located (gray) and 3D models of the designed scaffolds (blue), as seen in the same 3D space from different viewing points.

Figure 30 shows the 3D model of the part of the alveolar bone and the teeth where the periodontal lesions are focused, without the 3D models of the two scaffolds designed (left), as well as with the 3D models of the two scaffolds (right). The 3D model of the part of the alveolar bone and teeth where the periodontal lesions are focused and the 3D models of the two designed scaffolds are depicted in the same 3D space, from different viewpoints, in Figure 31.

In conclusion, the extraction socket preservation customized graft is less complex to model than the periodontal defect customized block graft, as its outer surfaces can be more easily designed through hole-filling procedures.

**Figure 32:**
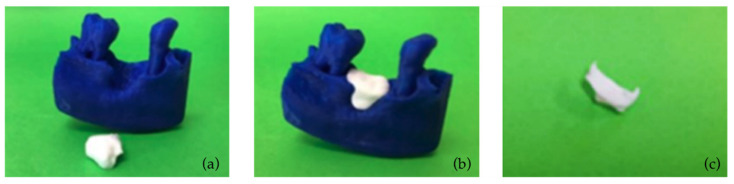
(**a**,**b**) 3D-printed models of the periodontal defect and the extraction socket preservation customized graft; (**c**) 3D-printed model of the periodontal defect customized block graft.

Both grafts and the hard tissues around the periodontal defects (alveolar bone and teeth) were 3D-printed using a FlashForge Creator Pro Fused Deposition Modeling (FDM) 3D printer (Zhejiang Flashforge 3D Technology Co., Jinhua, China), as illustrated in Figure 32. More information on 3D printing materials and settings may be found in [53].

## 4. Discussion

The treatment of osseous defects around teeth is a core issue of an entire dental specialty: Periodontology. These defects are the result of long-term gingival and periodontal tissue inflammation with various types of bacteria, leading to bone loss around teeth and compromised support of teeth. The goal of periodontal treatment is to eliminate the causative factors and, if possible, to allow bone regeneration to support the tooth structures. Apart from surgical procedures (scaling and root planning of teeth), medication delivery for osseous defects and grafting have been utilized over the years for the treatment of osseous defects. Grafting (placing donor bone tissue in the osseous defects) aims to fill the osseous cavity (defect) around teeth with some type of material or tissue in order to allow new bone to be formed by the patient. In a way, the grafting material simply acts as a scaffold “keeping the space” for the new bone to be formed by blocking soft tissues (gingivae) to fill in the osseous defect. A crucial factor affecting the success of this procedure is the contact interface of the grafting material to the defect (in a way, if it fills the defect without any gaps). This explains the need for custom-generated scaffolds, which match the anatomical details of the osseous cavity to be filled without leaving behind possible gaps, which may allow soft tissue proliferation.

In order to design such a scaffold, one needs to have in hand a 3D representation of the osseous defect with maximum possible detail, so that the resulting graft design will match the anatomy of the osseous defect as close to ideal as possible.

The aim of this article was to establish and evaluate a workflow for designing customized 3D scaffolds for personalized regenerative treatment against periodontitis. The proposed method relies on a CBCT-based 3D modeling procedure for acquiring the 3D morphology of the periodontal defect. Two types of methodology are outlined for the design of 3D periodontal scaffolds, i.e., for designing periodontal defect customized block grafts and extraction socket preservation customized grafts. The proposed workflow is easy to implement and may yield a highly accurate 3D model of a periodontal scaffold in less than two hours using CBCT data, which makes it a cost-effective solution for generating very detailed digital 3D models of grafts that may be 3D-printed. The proposed methodology has been successfully employed to generate 3D models of the hard tissues surrounding periodontal defects and to design patient-specific scaffolds for periodontal regeneration. These results will serve as the foundation for the 3D printing of bioabsorbable scaffolds for the personalized treatment of periodontitis, which can also serve as sustained-release drug carriers.

The Innovative elements of the research presented in this article are the following:

- The evaluation of the methodology proposed in our previous research work [49] for generating a 3D model of the hard tissues of a patient suffering from periodontitis, using the patient’s CBCT scan.
- The establishment of two workflows for designing two types of 3D periodontal scaffolds for personalized treatment against periodontitis.
- The evaluation of the aforementioned workflows for the generation of a periodontal defect customized block graft and an extraction socket preservation customized graft.

The grafts generated using the proposed 3D design process differ from the great majority of 3D-printed scaffolds reported in the literature, such as those ones presented in [27,28,29], because the latter scaffolds are either predesigned or have a standard structure, without taking into account any complex morphology of the real damage. On the other hand, both grafts created using the proposed workflow are patient-specific bone grafts, exactly fitted to the hard tissues of the patient’s periodontal defect. In spite of the fact that a few limited research articles tackle the issue of patient-specific scaffolds using CBCT data, such as [46,47,48], they do not reveal any technical details of the scaffold-designing process, which our article emphasizes, with the aim to establish an optimal workflow that may be followed for patient-specific scaffold design. What is more, the novelty of our design relies on the fact that with the reported workflow, one is able to represent with a great deal of detail osseous defects fine in dimensions and, thus, to design scaffolds that match the defects with high accuracy. Other similar processing algorithms may present adequate detail but for gross anatomical models, rather than fine ones such as the periodontal bone defects. Thus, this research will stimulate further investigation and clinical applications on this matter.

This research has been conducted in the context of the research project 3D-BioPerioDontis (“Bioresorbable Three-Dimensional (3D) Printed Scaffolds for Personalized Treatment of Periodontitis”) [54]. Thoughts for future research in the fields of CBCT-based 3D modeling, 3D printing, and dental use of the designed grafts concern:

- The further investigation, development, and application of tomographic image segmentation algorithms, in order to propose more automated 3D modeling methodologies using CBCT data.
- The comparison of Fused Deposition Modeling (FDM) and Selective Laser Sintering (SLS) as suitable 3D printing techniques for the designed bone grafts.
- The use of new biodegradable materials for 3D printing of the scaffolds, which will also act as carriers of drugs in patients.
- The clinical trials of surgical placement of the 3D-printed bone grafts in patients.

Albeit actual patient CBCT radiological data were used for the realistic representation of the anatomical models of the osseous defects, we had no way of comparing the accuracy of this representation to the gold standard (the appearance of the osseous defects and relevant models of the actual patients). This stands as the most significant limitation of the study. Our goal was the development and implementation of an image processing and 3D design workflow (algorithm) in order to achieve as realistic a representation of the osseous defects under investigation as possible. Our research team is already in collaboration with a team of clinical researchers in order to evaluate the accuracy of the resulting models and scaffolds to true patient scenarios. Finally, as biotechnology evolves and advances, we anticipate that ethical and regulatory issues will also be addressed.

## Figures and Tables

**Table 1:** Number of triangles and size of the generated 3D models of hard tissues around the periodontal defects.

3D Model	Number of Triangles	Size (MB)
Teeth (before processing)	156,852	7.7
Alveolar bone (before processing)	608,144	26.7
Teeth and alveolar bone (after processing)	509,518	24.9

## Data Availability

Not applicable.

## References

[1] Aimar A., Palermo A., Innocenti B. (2019). The Role of 3D Printing in Medical Applications: A State of the Art. J. Healthc. Eng..

[2] Verykokou S., Ioannidis C. (2023). An Overview on Image-Based and Scanner-Based 3D Modeling Technologies. Sensors.

[3] Do A.-V., Khorsand B., Geary S.M., Salem A.K. (2015). 3D Printing of Scaffolds for Tissue Regeneration Applications. Adv. Healthc. Mater..

[4] Zhang L., Yang G., Johnson B.N., Jia X. (2019). Three-Dimensional (3D) Printed Scaffold and Material Selection for Bone Repair. Acta Biomater..

[5] Varma M.V., Kandasubramanian B., Ibrahim S.M. (2020). 3D Printed Scaffolds for Biomedical Applications. Mater. Chem. Phys..

[6] Bahraminasab M. (2020). Challenges on Optimization of 3D-Printed Bone Scaffolds. BioMed. Eng. Online.

[7] Kanwar S., Vijayavenkataraman S. (2021). Design of 3D Printed Scaffolds for Bone Tissue Engineering: A Review. Bioprinting.

[8] Yadav L.R., Chandran S.V., Lavanya K., Selvamurugan N. (2021). Chitosan-Based 3D-Printed Scaffolds for Bone Tissue Engineering. Int. J. Biol. Macromol..

[9] Wang Z., Wang Y., Yan J., Zhang K., Lin F., Xiang L., Deng L., Guan Z., Cui W., Zhang H. (2021). Pharmaceutical Electrospinning and 3D Printing Scaffold Design for Bone Regeneration. Adv. Drug Deliv. Rev..

[10] Ansari M.A.A., Golebiowska A.A., Dash M., Kumar P., Jain P.K., Nukavarapu S.P., Ramakrishna S., Nanda H.S. (2022). Engineering Biomaterials to 3D-Print Scaffolds for Bone Regeneration: Practical and Theoretical Consideration. Biomater. Sci..

[11] Karanth D., Song K., Martin M.L., Meyer D.R., Dolce C., Huang Y., Holliday L.S. (2023). Towards Resorbable 3D-printed Scaffolds for Craniofacial Bone Regeneration. Orthod. Craniofacial Res..

[12] Mirkhalaf M., Men Y., Wang R., No Y., Zreiqat H. (2023). Personalized 3D Printed Bone Scaffolds: A Review. Acta Biomater..

[13] Ma H., Feng C., Chang J., Wu C. (2018). 3D-Printed Bioceramic Scaffolds: From Bone Tissue Engineering to Tumor Therapy. Acta Biomater..

[14] Tian Y., Chen C., Xu X., Wang J., Hou X., Li K., Lu X., Shi H., Lee E.-S., Jiang H.B. (2021). A Review of 3D Printing in Dentistry: Technologies, Affecting Factors, and Applications. Scanning.

[15] Habib A.A.I., Sheikh N.A. (2022). 3D Printing Review in Numerous Applications for Dentistry. J. Inst. Eng. India Ser. C.

[16] Larsson L., Decker A.M., Nibali L., Pilipchuk S.P., Berglundh T., Giannobile W.V. (2016). Regenerative Medicine for Periodontal and Peri-Implant Diseases. J. Dent. Res..

[17] Asa’ad F., Pagni G., Pilipchuk S.P., Giannì A.B., Giannobile W.V., Rasperini G. (2016). 3D-Printed Scaffolds and Biomaterials: Review of Alveolar Bone Augmentation and Periodontal Regeneration Applications. Int. J. Dent..

[18] Louvrier A., Marty P., Barrabé A., Euvrard E., Chatelain B., Weber E., Meyer C. (2017). How Useful Is 3D Printing in Maxillofacial Surgery?. J. Stomatol. Oral Maxillofac. Surg..

[19] Oberoi G., Nitsch S., Edelmayer M., Janjić K., Müller A.S., Agis H. (2018). 3D Printing—Encompassing the Facets of Dentistry. Front. Bioeng. Biotechnol..

[20] Yu N., Nguyen T., Cho Y.D., Kavanagh N.M., Ghassib I., Giannobile W.V. (2019). Personalized Scaffolding Technologies for Alveolar Bone Regenerative Medicine. Orthod. Craniofac Res..

[21] Lin L., Fang Y., Liao Y., Chen G., Gao C., Zhu P. (2019). 3D Printing and Digital Processing Techniques in Dentistry: A Review of Literature. Adv. Eng. Mater..

[22] Huang M.F., Alfi D., Alfi J., Huang A.T. (2019). The Use of Patient-Specific Implants in Oral and Maxillofacial Surgery. Oral Maxillofac. Surg. Clin. N. Am..

[23] Sharma D., Mathur V.P., Satapathy B.K. (2021). Biodegradable and Biocompatible 3D Constructs for Dental Applications: Manufacturing Options and Perspectives. Ann. Biomed. Eng..

[24] Iranmanesh P., Ehsani A., Khademi A., Asefnejad A., Shahriari S., Soleimani M., Ghadiri Nejad M., Saber-Samandari S., Khandan A. (2022). Application of 3D Bioprinters for Dental Pulp Regeneration and Tissue Engineering (Porous Architecture). Transp. Porous Med..

[25] Sikdar R., Bag A., Shirolkar S., Gayen K., Sarkar S., Roychowdhury S. (2022). 3D Printing: Its Application in Pediatric Dental Practice. Acta Sci. Dent. Sci..

[26] Jain P., Kathuria H., Dubey N. (2022). Advances in 3D Bioprinting of Tissues/Organs for Regenerative Medicine and in-Vitro Models. Biomaterials.

[27] Park J., Lee S.J., Jo H.H., Lee J.H., Kim W.D., Lee J.Y., Park S.A. (2017). Fabrication and Characterization of 3D-Printed Bone-like β-Tricalcium Phosphate/Polycaprolactone Scaffolds for Dental Tissue Engineering. J. Ind. Eng. Chem..

[28] Buyuksungur S., Hasirci V., Hasirci N. (2021). 3D Printed Hybrid Bone Constructs of PCL and Dental Pulp Stem Cells Loaded GelMA. J. Biomed. Mater. Res..

[29] Zhang C., Chen Z., Liu J., Wu M., Yang J., Zhu Y., Lu W.W., Ruan C. (2022). 3D-Printed Pre-Tapped-Hole Scaffolds Facilitate One-Step Surgery of Predictable Alveolar Bone Augmentation and Simultaneous Dental Implantation. Compos. Part B Eng..

[30] Staples R.J., Ivanovski S., Vaquette C. (2020). Fibre Guiding Scaffolds for Periodontal Tissue Engineering. J. Periodont Res..

[31] Wang C.-Y., Chiu Y.-C., Lee A.K.-X., Lin Y.-A., Lin P.-Y., Shie M.-Y. (2021). Biofabrication of Gingival Fibroblast Cell-Laden Collagen/Strontium-Doped Calcium Silicate 3D-Printed Bi-Layered Scaffold for Osteoporotic Periodontal Regeneration. Biomedicines.

[32] Lin H.-H., Chao P.-H.G., Tai W.-C., Chang P.-C. (2021). 3D-Printed Collagen-Based Waveform Microfibrous Scaffold for Periodontal Ligament Reconstruction. Int. J. Mol. Sci..

[33] Lee U.-L., Yun S., Cao H.-L., Ahn G., Shim J.-H., Woo S.-H., Choung P.-H. (2021). Bioprinting on 3D Printed Titanium Scaffolds for Periodontal Ligament Regeneration. Cells.

[34] Park J., Park S., Kim J.E., Jang K.-J., Seonwoo H., Chung J.H. (2021). Enhanced Osteogenic Differentiation of Periodontal Ligament Stem Cells Using a Graphene Oxide-Coated Poly(ε-Caprolactone) Scaffold. Polymers.

[35] Sufaru I.-G., Macovei G., Stoleriu S., Martu M.-A., Luchian I., Kappenberg-Nitescu D.-C., Solomon S.M. (2022). 3D Printed and Bioprinted Membranes and Scaffolds for the Periodontal Tissue Regeneration: A Narrative Review. Membranes.

[36] Li C., Xu X., Gao J., Zhang X., Chen Y., Li R., Shen J. (2022). 3D Printed Scaffold for Repairing Bone Defects in Apical Periodontitis. BMC Oral Health.

[37] Yao Y., Raymond J.E., Kauffmann F., Maekawa S., Sugai J.V., Lahann J., Giannobile W.V. (2022). Multicompartmental Scaffolds for Coordinated Periodontal Tissue Engineering. J. Dent. Res..

[38] Alhroob K.H., Alsabbagh M.M., Alsabbagh A.Y. (2021). Effect of the Use of a Surgical Guide on Heat Generation during Implant Placement: A Comparative in Vitro Study. Dent. Med. Probl..

[39] Paradowska-Stolarz A., Malysa A., Mikulewicz M. (2022). Comparison of the Compression and Tensile Modulus of Two Chosen Resins Used in Dentistry for 3D Printing. Materials.

[40] AlJehani Y.A. (2014). Diagnostic Applications of Cone-Beam CT for Periodontal Diseases. Int. J. Dent..

[41] Bagis N., Kolsuz M.E., Kursun S., Orhan K. (2015). Comparison of Intraoral Radiography and Cone-Beam Computed Tomography for the Detection of Periodontal Defects: An in Vitro Study. BMC Oral Health.

[42] Cetmili H., Tassoker M., Sener S. (2019). Comparison of Cone-Beam Computed Tomography with Bitewing Radiography for Detection of Periodontal Bone Loss and Assessment of Effects of Different Voxel Resolutions: An in Vitro Study. Oral Radiol..

[43] Zhang X., Li Y., Ge Z., Zhao H., Miao L., Pan Y. (2020). The Dimension and Morphology of Alveolar Bone at Maxillary Anterior Teeth in Periodontitis: A Retrospective Analysis-Using CBCT. Int. J. Oral Sci..

[44] Rinne C.A., Dagassan-Berndt D.C., Connert T., Müller-Gerbl M., Weiger R., Walter C. (2020). Impact of CBCT Image Quality on the Confidence of Furcation Measurements. J. Clin. Periodontol..

[45] Rasperini G., Pilipchuk S.P., Flanagan C.L., Park C.H., Pagni G., Hollister S.J., Giannobile W.V. (2015). 3D-Printed Bioresorbable Scaffold for Periodontal Repair. J. Dent. Res..

[46] Korn P., Ahlfeld T., Lahmeyer F., Kilian D., Sembdner P., Stelzer R., Pradel W., Franke A., Rauner M., Range U. (2020). 3D Printing of Bone Grafts for Cleft Alveolar Osteoplasty—In Vivo Evaluation in a Preclinical Model. Front. Bioeng. Biotechnol..

[47] Anderson M., Dubey N., Bogie K., Cao C., Li J., Lerchbacker J., Mendonça G., Kauffmann F., Bottino M.C., Kaigler D. (2022). Three-Dimensional Printing of Clinical Scale and Personalized Calcium Phosphate Scaffolds for Alveolar Bone Reconstruction. Dent. Mater..

[48] Schulz M.C., Holtzhausen S., Nies B., Heinemann S., Muallah D., Kroschwald L., Paetzold-Byhain K., Lauer G., Sembdner P. (2023). Three-Dimensional Plotted Calcium Phosphate Scaffolds for Bone Defect Augmentation—A New Method for Regeneration. J. Pers. Med..

[49] Verykokou S., Ioannidis C., Angelopoulos C. (2022). Evaluation of 3D Modeling Workflows Using Dental CBCT Data for Periodontal Regenerative Treatment. J. Pers. Med..

[50] 3D Slicer Image Computing Platform. https://www.slicer.org/.

[51] Geomagic Wrap. https://www.artec3d.com/3d-software/geomagic-wrap.

[52] Zhu L., Kolesov I., Gao Y., Kikinis R., Tannenbaum A. An Effective Interactive Medical Image Segmentation Method Using Fast GrowCut. Proceedings of the 17th International Conference on Medical Image Computing and Computer Assisted Intervention (MICCAI).

[53] Kapourani A., Koromili M., Verykokou S., Angelopoulos C., Ioannidis C., Valkanioti V., Chatzitheodoridou M., Barmpalexis P. Comparison of FDM and SLS printing of periodontal scaffolds using 3D models derived from CBCT scans. Proceedings of the 4th European Conference on Pharmaceutics.

[54] 3D-BioPerioDontis. https://3dperiodontis.gr/.

